# Exploring the Pattern Associated With Longitudinal Changes of β-Amyloid Deposition During Cognitively Normal Healthy Aging

**DOI:** 10.3389/fmed.2020.617173

**Published:** 2021-01-13

**Authors:** Yunyan Xie, Qin Yang, Chunhua Liu, Qi Zhang, Jiehui Jiang, Ying Han, The Alzheimer's Disease Neuroimaging Initiative

**Affiliations:** ^1^Department of Neurology, Xuanwu Hospital of Capital Medical University, Beijing, China; ^2^Key Laboratory of Specialty Fiber Optics and Optical Access Networks, Joint International Research Laboratory of Specialty Fiber Optics and Advanced Communication, School of Communication and Information Technology, Shanghai University, Shanghai, China; ^3^Shanghai Institute for Advanced Communication and Data Science, Shanghai University, Shanghai, China; ^4^Institute of Biomedical Engineering, Shanghai University, Shanghai, China; ^5^Center of Alzheimer's Disease, Beijing Institute for Brain Disorders, Beijing, China; ^6^National Clinical Research Center for Geriatric Disorders, Beijing, China

**Keywords:** healthy aging, ^18^F-AV-45 PET, β-amyloid deposition, brain, pattern

## Abstract

The aim of this study was to determine a pattern associated with longitudinal changes of β-amyloid (Aβ) deposition during cognitively normal(CN) healthy aging. We used ^18^F-florbetapir (AV-45) PET images of the brains of 207 cognitively normal subjects (CN1), obtained through the Alzheimer's Disease Neuroimaging Initiative (ADNI), to identify the healthy aging pattern and 76 cognitively normal healthy subjects (CN2), obtained through the Xuanwu Hospital of Capital Medical University, Beijing, China, to verify it. A voxel-based correlation analysis of standardized uptake value ratio (SUVR) map image and age was conducted using the DPABI (Data Processing & Analysis of Brain Imaging) software to identify the pattern. The sum of squares due to errors (SSE), R-square (R^2^) and the root-mean-square error (RMSE) were calculated to assess the quality of curve fitting. Among them, R^2^ was proposed as the coherence coefficient, which was as an index to assess the correlation between SUVR value of the pattern and subjects' age. The pattern characterized by age-associated longitudinal changes of Aβ deposition was mainly distributed in the right middle and inferior temporal gyrus, the right temporal pole: middle temporal gyrus, the right inferior occipital gyrus, the right inferior frontal gyrus (triangular portion), and the right precentral gyrus. There were a significant positive correlation between the SUVR value of the pattern and age for each CN group (CN1: R^2^ = 0.120, *p* < 0.001 for quadratic model; CN2: R^2^ = 0.152, *p* = 0.002 for quadratic model). These findings suggest a pattern of changes in Aβ deposition that can be used to distinguish physiological changes from pathophysiological changes, constituting a new method for elucidating the neuropathological mechanism of Alzheimer's disease.

## Introduction

Brain aging, which is influenced by various pathological and psychosocial factors (1), comprises two categories: healthy and pathological aging. According to clinical neurology, healthy aging is defined as “*the cognitively normal (CN) subjects who maintain their normal cognitive level and ability of daily living as they grow older, without neurological diseases*” (2). Pathological aging, which is characterized by the accumulation of extracellular Aβ deposition (3), is considered a major pathological element of Alzheimer's disease (AD) (4). However, the presence of Aβ in the AD brain may also signal a physiological age-associated phenomenon depending on its extent and distribution pattern (5–8). Research evidence suggests that Aβ deposition occurs in the brains of cognitively normal older individuals (9–12). The prevalence of the amyloid burden among cognitively normal older individuals has been estimated to be more than 25% than younger individuals based on the findings of autopsy studies (9, 13, 14). However, given limited knowledge regarding the extent and distribution of Aβ deposition during the healthy aging process, an assessment of changes in Aβ deposition with age is essential for advancing understanding of healthy aging.

Some studies that have measured amyloid deposition in the course of normal aging found a significant linear increase in global Aβ deposition with age (3, 12, 15). One study found a highly significant correlation between increasing age and a reduction in Aβ turnover rates (16). Significant linear increases with age have been observed in the precuneus, temporal cortex, and the anterior and posterior cingulate (3) as well as in the frontal, cingulate and parietal areas, with primary sensory/visual areas being relatively protected from amyloid deposition (17). The findings of the above studies indicate that there may be a linear pattern of brain aging associated with changes in Aβ deposition during healthy aging in cognitively normal adults. However, all above studies were based on western datasets and the repeatability of results was not verified among different ethnic cohorts.

^18^F-florbetapir (AV-45) is a safe tracer demonstrating high levels of sensitivity and specificity for Aβ detection (18). Aβ deposition in the brain can be quantified within a clinical environment through positron emission computed tomography (PET) scans conducted with ^18^F-AV-45 (19). Moreover, this technique can be used to study Aβ distribution *in vivo*, enabling the formation and progression of Aβ aggregates in the brain to be monitored (20, 21). Thus, there were two main objectives in this study: (1) to explore a pattern associated with longitudinal changes of Aβ deposition during healthy aging using ^18^F-AV-45 PET images to quantify Aβ deposition *in vivo*. (2) to verify the repeatability of the healthy aging pattern among western and Chinese cohorts.

## Materials and Methods

### Materials

Two cohorts of neuroimaging data were collected from two independent centers: Cohort A (N = 207, right-handed, CN1) from the Alzheimer's Disease Neuroimaging Initiative (ADNI) database (http://adni.loni.usc.edu/) and cohort B (N = 76, right-handed, CN2) from the Department of Neurology, Xuanwu Hospital of Capital Medical University. Both clinical (Sex, Age, Education, Mini-Mental State Examination (MMSE)) and image (18F-AV-45 PET and MRI image) information were selected for the two cohorts. Montreal Cognitive Assessment (MoCA) and Clinical Dementia Rating Sum of Boxes (CDR-SB) were also selected for Cohort A. Notablly, in total 378 images were included for Cohort A because part of subjects had more than one scan (1.83 ± 0.83 scan times per subject), while each subject had only one scan in Cohort B.

**Figure 1:**
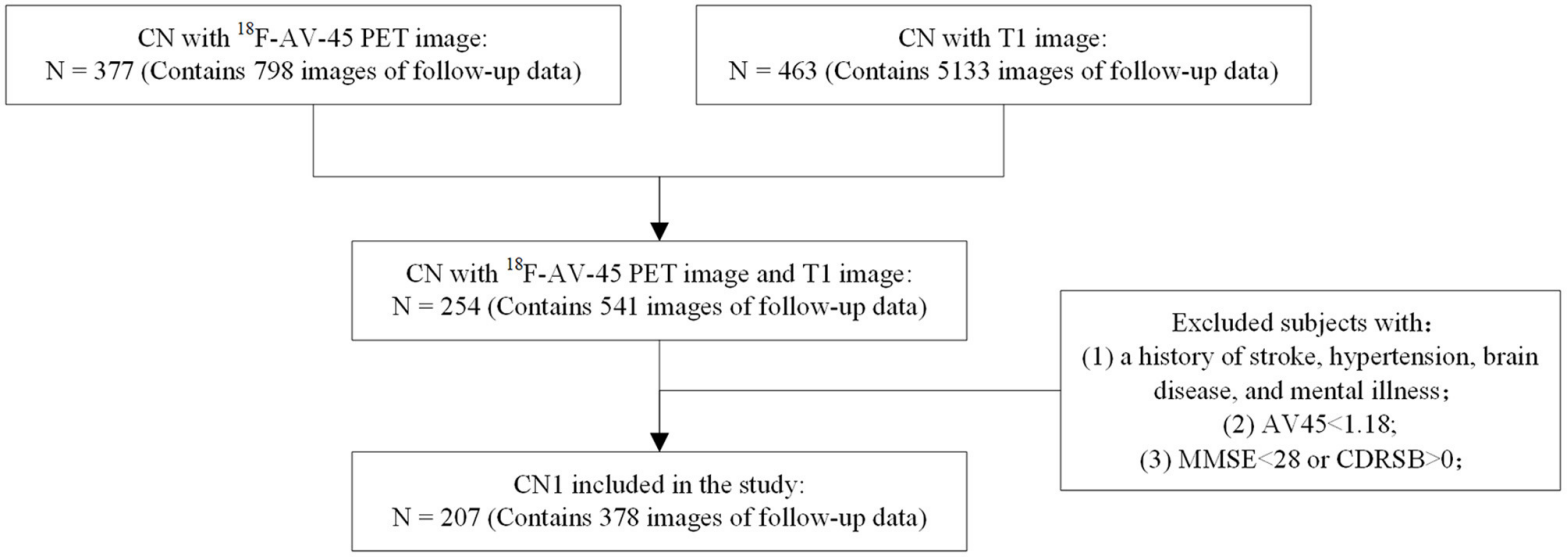
Inclusion and exclusion criteria applied to the ADNI data.

Figure 1 shows the exclusion and inclusion criteria applied to CN subjects of Cohort A. The following inclusion criteria were applied: (1) subjects had no history of stroke, hypertension, brain disease, or mental illness. (2) The PET scan results of individuals were Aβ-negative (Aβ-), with a cerebral-to-whole cerebellar florbetapir SUVR value below 1.18 (22). (3) Mini-Mental State Examination (MMSE) scores for individuals were above or equal to 28, and their Clinical Dementia Rating Sum of Boxes (CDR-SB) scores were all 0. Inclusion criteria for subjects in Cohort B were consistent with those for Cohort A. This study was approved by the institutional review boards of ADNI and the Research Ethics Committee of Xuanwu Hospital, Beijing, China.Written, informed consent had been obtained from each subject.

### Image Acquisition Protocol

The process of acquiring data for the CN1 group is described in detail in the imaging protocol column of the ADNI database (http://adni.loni.usc.edu/). PET and T1 MRI data were simultaneously obtained for each participant in the CN2 group. All of the participants were invited to undergo optional ^18^F-florbetapir (AV-45) PET scans in the three-dimensional acquisition mode. A dynamic scan, lasting 35 min, was performed approximately 40 min after participants received an intravenous injection of 7–10 mCi [^18^F] florbetapir. The PET scan images were analytically reconstructed using a time-of-flight ordered subset expectation maximization (TOF OSEM) algorithm with the following parameters: eight iterations, 32 subsets matrix = 192 × 192, field of view (FOV) = 350 × 350, half-width height = 3.

Three-dimensional T1-weighted magnetization-prepared rapid gradient echo scans were performed using an integrated TOF-capable PET/MR 3.0T imaging device (SIGNA PET/MR, GE Healthcare, Milwaukee, Wisconsin, USA) available at the Xuanwu Hospital of Capital Medical University. The following parameters were applied: SPGR sequence, FOV = 256 × 256 mm^2^, matrix = 256 × 256, slice thickness = 1 mm, gap = 0, slice number = 192, repetition time (TR) = 6.9 ms, echo time (TE) = 2.98 ms, inversion time (TI) = 450 ms, flip angle = 12°, voxel size = 1 × 1 × 1 mm^3^.

### Image Preprocessing

All ^18^F-AV-45 PET scan images and corresponding T1 images were preprocessed using statistical parametric mapping software (SPM12; https://www.fil.ion.ucl.ac.uk/spm/software/spm12/) in MATLAB (Version R2014a; MathWorks, Natick, MA, United States). We first used the realigning method to ensure that all frames in the dynamic scans were motion-corrected to the first frame and processed the output single average functional image, reducing system, or head motion errors. Next, we performed a voxel-based partial volume effect (PVE) correction of the functional image using the Müller-Gärtner method (MG), with parameters of white matter (WM), gray matter (GM), and cerebrospinal fluid (CSF) obtained through T1 image segmentation. Then, PVE-corrected image was then normalized with reference to the standard Montreal Neurological Institute (MNI) brain space using the deformation field from the MRI image to the MNI space and smoothed to reduce noise and improve image quality using an isotropic Gaussian smoothing kernel with a gaussian filter of 8 mm full-width at half-maximum (FWHM). Lastly, the smoothed functional image was intensity normalized to the mean uptake of whole cerebellum to obtain SUVR map image.

### Voxel-Wise PET Analysis

To explore the effect of age on Aβ deposition in the brains of cognitively normal subjects, a voxel-wise correlation analysis of SUVR map iamges was conducted for CN1, with age applied as the seed series and GM, sex, and years of education considered as the covariates. The DPABI software in MATLAB R2014a was used for the analysis. Accordingly, we obtained a statistical map (false discovery rate (FDR) corrected with q < 0.01) reflecting the change trend and degree of Aβ deposition in the aging brain. Thereby voxels relating to aging were obtaining with the absolute value of the correlation coefficient ≥ 0.3. As a final step, we mapped the voxels on to the MNI standard space to obtain statistical brain regions as ROIs. To verify that ROIs actually reflect the effect of aging on Aβ deposition in cognitively normal individuals, we examined the correlations between the SUVR values of the healthy aging pattern and age for individuals in the CN1 group and compared the results with the SUVR value for the whole brain. SUVR values were plotted against subjects' ages and fitted using three separate models, namely a linear model:

(1) $$\begin{array}{r} \text{y}=\text{a}t+\text{b}, \end{array}$$

a quadratic model:

(2) $$\begin{array}{r} \text{y}=\text{a}t^{2}+\text{b}t+\text{c}, \end{array}$$

and an exponential model:

(3) $$\begin{array}{r} \text{y}=\text{a}e^{\text{b}t}+\text{c}, \end{array}$$

where *t* denotes age and a, b, and c are the parameters to be estimated from the data plotted for the SUVR values and ages of subjects in the CN1 group. The sum of squares due to errors (SSE), R-square (R^2^), and the root-mean-square error (RMSE) values were calculated to assess the quality of fit. Among them, R^2^ was proposed as the coherence coefficient, which was as an index to assess the correlation between SUVR value of the pattern and subjects' age. Subsequently, the model with the best quality of fit was assigned to the plotted SUVR and age obtained for the CN1 group to evaluate the change trend of Aβ deposition with aging. Forward validation was performed on the CN2 group.

### Statistical Analysis

The quantitative results obtained with MATLAB were subjected to a statistical analysis using the SPSS software, version 18.0 (SPSS Inc., IBM Corporation, Chicago, USA). A two-sample t-test was performed to examine differences in continuous variables, and a Chi-square test was conducted to assess categorical variables. *p* < 0.05 was considered statistically significant. The Gramm toolbox in MATLAB was used for plotting and visualizing all of the statistical data presented in this paper (23).

## Results

### Demographic Characteristics of the Participants

Cohort A contained 378 time ponts of 207 CN subjects (1.83 ± 0.83 time points per subject). Cohort B included 76 time poits of 76 CN subjects (one time point per subject). Table 1 shows the demographic and clinical details of the two cohorts. As shown in Table 1, significant differences between cohort A and cohort B are observed in age (*p* < 0.001), sex (*p* = 0.0488) and education (*p* < 0.001). A slight difference is observed in sex(*p* = 0.049) and no significant difference in MMSE (*p* = 0.119). Considering the impact of brain atrophy and differences relating to the sex and education levels of the participants, we reported the results obtained after regressing the covariates of GM, sex, and the number of years of education.

**Table 1:** Demographic and clinical characteristics of participants.

	**CN1**	**CN2**	***P*-value**
N	378	76	–
Age (years)	74.8 ± 5.6	65.2 ± 5.2	<0.001
Sex (F/M)	192/186	48/28	0.049
Education (years)	16.6 ± 2.5	12.8 ± 3.4	<0.001
MMSE	29.4 ± 0.8	29.2 ± 0.7	0.119
MoCA	26.2 ± 2.3	–	–
CDR-SB	0.0 ± 0.0	–	–

*MMSE, mini-mental state examination; MoCA, montreal cognitive assessment; CDR-SB, clinical dementia rating sum of boxes*.

### Voxel-Wise PET Analysis

#### Healthy Aging Pattern

**Figure 2:**
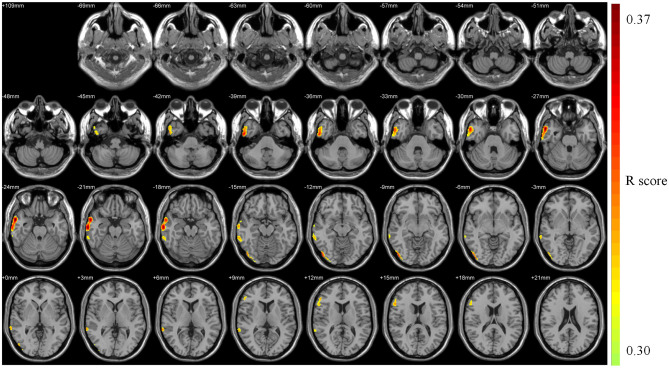
Pattern associated with longitudinal changes of Aβ deposition in cognitively normal adults (CN1) during the healthy aging. The red areas are those with significant increases in Aβ deposition associated with advancing age. No voxels were found that indicated significant decreases in Aβ deposition associated with advancing age.

The health aging pattern was identified in CN1 group. The results of the correlation analysis revealed that there was a healthy aging pattern characterized by age-associated longitudinal changes of Aβ deposition was mainly distributed in the right middle and inferior temporal gyrus, the right temporal pole: middle temporal gyrus, the right inferior occipital gyrus, the right inferior frontal gyrus (triangular portion), and the right precentral gyrus (Figure 2). No areas of the brain evidenced significantly decreased Aβ deposition (see Table 2 for details).

**Table 2:** Pattern associated with longitudinal changes of Aβ deposition in cognitively normal adults (CN1) during healthy aging.

**Region**	**Laterality**	**Cluster extent**	**Peak Voxel**			
			**T**	**X**	**Y**	**Z**
Middle temporal gyrus	Right	870	0.37	58	4	−24
Inferior temporal gyrus	Right					
Temporal pole: middle temporal gyrus	Right					
Middle temporal gyrus	Right	229	0.33	68	−42	6
Inferior temporal gyrus	Right					
Inferior occipital gyrus	Right	159	0.34	48	−82	−10
Inferior frontal gyrus (triangular portion)	Right	117	0.34	56	20	14
Precentral gyrus	Right	133	0.34	54	10	30

#### Pattern Validation

**Figure 3:**
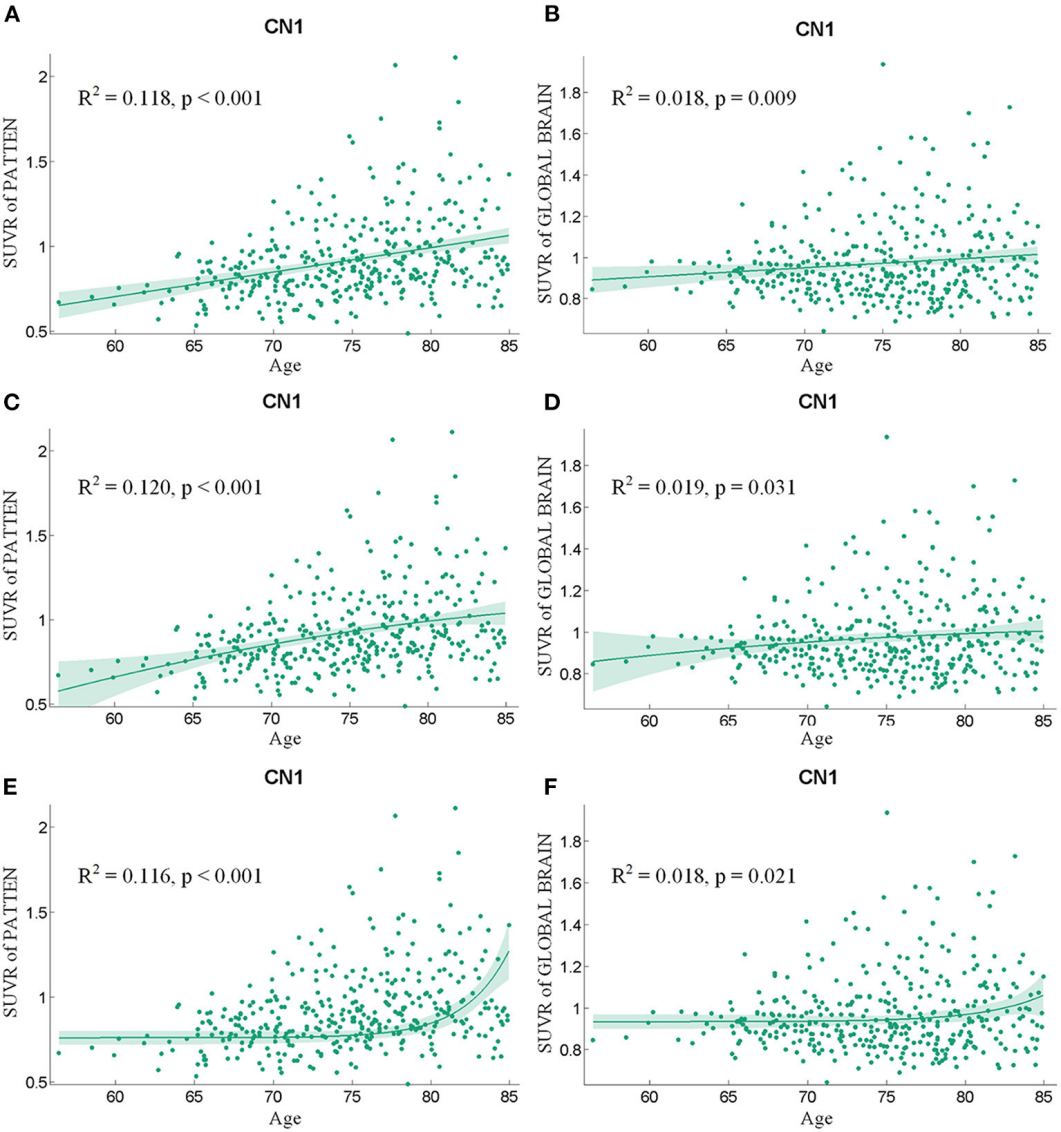
The findings of a correlation analysis of age and the SUVR values of the pattern **(A,C,E)** and the whole brain **(B,D,F)** of individuals in the CN1 group using linear, quadratic, and exponential model fitting, respectively.

The health aging pattern was further validated in CN2 group. Following the regression of the covariates of GM, sex, and years of education, SUVR value of the pattern showed a significant positive correlation with age (Figure 3), whereas SUVR value of global brain showed a weaker positive correlation with age (Figure 3) in the CN1 group. Table 3 shows the curve fit results for the SUVR of the pattern and age of the three models. Specific results were as follows: SSE = 18.549, R^2^ = 0.118, and RMSE = 0.222 for the linear model; SSE = 18.505, R^2^ = 0.120, and RMSE = 0.222 for the quadratic model; and SSE = 18.592, R^2^ = 0.116, and RMSE = 0.222 for the exponential model. The curve fit results for the SUVR of global brain and age for the three models were as follows: SSE = 12.459, R^2^ = 0.018, and RMSE = 0.182 for the linear model; SSE = 12.452, R^2^ = 0.019, and RMSE = 0.182 for the quadratic model; and SSE = 12.461, R^2^ = 0.018 and RMSE = 0.182 for the exponential model.

**Table 3:** The curve-fitting characteristics of SUVR value and age for the CN1 group.

	**SUVR of the pattern**			**SUVR of global brain**		
	**Linear model**	**Quadratic model**	**Exponential model**	**Linear model**	**Quadratic model**	**Exponential model**
SSE	18.549	18.505	18.592	12.459	12.452	12.461
R^2^	0.118	0.120	0.116	0.018	0.019	0.018
RMSE	0.222	0.222	0.222	0.182	0.182	0.182
*p*-value	<0.001	<0.001	<0.001	0.009	0.031	0.021

*SSE, sum of squares due to errors; R^2^ R-square; RMSE, the root-mean-square error*.

**Figure 4:**
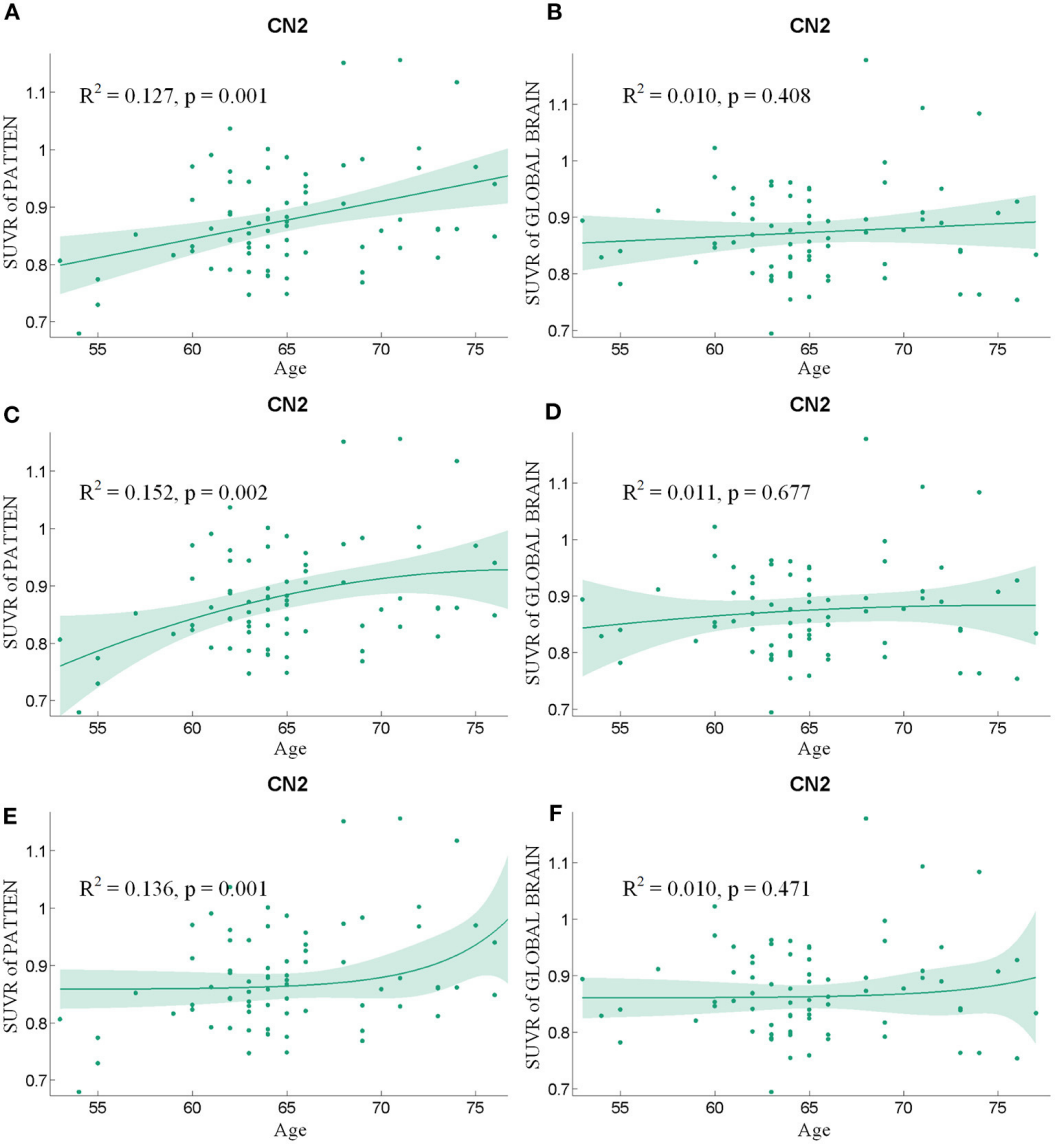
The findings of a correlation analysis of age and the SUVR values of the pattern **(A,C,E)** and the whole brain **(B,D,F)** of individuals in the CN2 group using linear, quadratic, and exponential model fitting respectively.

Following the regression of the covariates of GM, sex, and educational years, the SUVR value of the pattern showed a significant positive correlation with age (Figure 4), whereas SUVR value of global brain showed no significant correlation with age (Figure 4) in the CN2 group. Table 4 shows the curve fit results for SUVR of the pattern and age for the three models. Specific results were as follows: SSE = 0.534, R^2^ = 0.127 and RMSE = 0.085 for the linear model; SSE = 0.526, R^2^ = 0.152 and RMSE = 0.085 for the quadratic model; SSE = 0.535, R^2^ = 0.136, and RMSE = 0.085 for the exponential model. The results for the curve fit of the SUVR of global brain and age were as follows: SSE = 0.502, R^2^ = 0.010, and RMSE = 0.082 for the linear model; SSE = 0.501, R^2^ = 0.011, and RMSE = 0.083 for the quadratic model; and SSE = 0.502, R^2^ = 0.010, and RMSE = 0.082 for the exponential model.

**Table 4:** The curve-fitting characteristics of SUVR value and age for the CN2 group.

	**SUVR of the pattern**			**SUVR of global brain**		
	**Linear model**	**Quadratic model**	**Exponential model**	**Linear model**	**Quadratic model**	**Exponential model**
SSE	0.534	0.526	0.535	0.502	0.501	0.502
R^2^	0.127	0.152	0.136	0.010	0.011	0.010
RMSE	0.085	0.085	0.085	0.082	0.083	0.082
*p*-value	0.001	0.002	0.001	0.408	0.677	0.471

*SSE, sum of squares due to errors; R^2^ R-square; RMSE, the root-mean-square error*.

## Discussion

During the healthy aging of cognitively normal adults, SUVR value of a healthy aging pattern increased significantly. The pattern was mainly distributed in the right middle and inferior temporal gyrus, the right temporal pole: middle temporal gyrus, the right inferior occipital gyrus, the right inferior frontal gyrus (triangular portion), and the right precentral gyrus. A weak positive correlation was found between SUVR of global brain and age for the CN1 group, with no significant correlation existing for the CN2 group. These results indicate that during the healthy aging process of cognitively normal people, the increase in Aβ deposition is concentrated in specific brain regions rather than being distributed throughout the brain. In addition, the pattern shows a characteristic of asymmetric amyloid accumulation. Alteration in hemispheric asymmetry has been referenced in studies of healthy aging (24, 25). The Right hemi-aging model proposes that the right hemisphere presents greater aging than the left hemisphere (26, 27), so our results were consistent with previous studies.

The best fit curves for the SUVR value of the healthy aging pattern and age within each CN group reflected a change trend of increasing Aβ deposition on the pattern with increasing age and a subsequent decrease in the growth rate of Aβ deposition. This indicated significant changes in Aβ deposition in aging adults with normal cognition. However, when accumulated deposits of Aβ exceeded a certain threshold, leading to cognitive impairment, aging had weaker effect on Aβ deposition. This finding suggests that the baseline level of Aβ may differ for patients with AD. Moreover, as indicated by the findings of other studies, with the advancement of pathological conditions, Aβ deposition may reach a saturation point and will no longer exhibit a linear relationship with age (17, 28). This finding is supported by that of another study, which revealed that Aβ increases significantly in individuals with normal cognitive functions but that the rate of increase of Aβ slows down following the onset of cognitive impairment (16).

**Figure 5:**
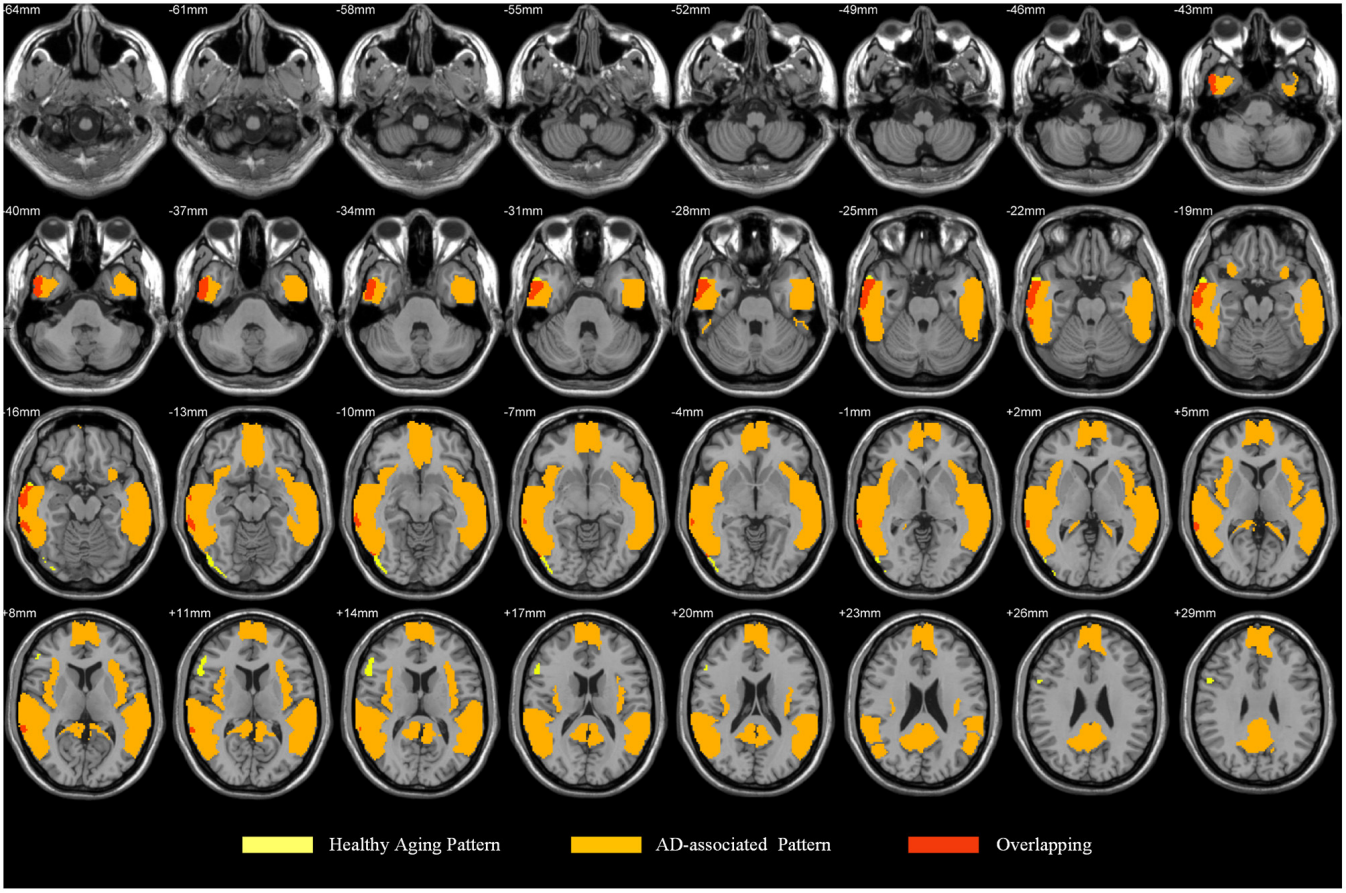
The overlap between our proposed pattern and AD parttern in previous studies.

Considering our results together with the findings reported in the literature, we posit that the healthy aging pattern associated with longitudinal changes of Aβ deposition and characteristic regions associated with AD partially overlap, mainly including the middle and inferior temporal gyrus (29, 30) (Figure 5). The temporal lobe and occipital cortex are associated with auditory and visual functions (31). The rapid deposition of Aβ in the middle and inferior occipital gyrus and in the middle and inferior temporal gyrus may be one of the reasons why the auditory and visual fields are influenced by age-associated and neurochemical factors (32) and may reflect a decline in the multisensory integration capacity of older individuals (33). It may account for the importance of age as an influencing factor affecting the diagnosis of AD or early AD.

Moreover, our results on age-associated changes of Aβ deposition could be explained from the perspective of molecular cytology. During the aging process, over-activated microglia may release neurotoxic molecules and pro-inflammatory cytokines, leading to neuronal death and inflammation and an accelerated process of Aβ deposition and accumulation (34, 35). Age-associated increases in microglial activation may contribute to the age-associated increase on Aβ deposition. Past research showed that significant age-associated increases in the total numbers of activated IL-1α+ microglia occurred in mesial temporal lobe (36). And the density of amyloid plaques in the temporal lobe is not related to memory level (37). Our findings on the healthy aging pattern were consistent with previous studies, suggesting that the increase in Aβ deposition promoted by normal aging in the temporal lobe were not caused from cognitive decline.

It should be noted that this study had some limitations. First, the datasets used for the study were limited. Although the pattern were identified and validated using data sourced from the ADNI and Xuanwu Hospital, multicenter research and autopsy results are required to confirm their universality. Second, age differences could be observed between the ADNI and Xuanwu Hospital in this study, and the average age of subjects from Xuanwu hospital was 9.4 years younger than ADNI. Although the correlation between SUVR of pattern and age were found in both cohorts, whether this correlation exsited in older Chinese CN population need be verified in the future. Third, this study was evidently a cross-sectional study, although follow-up data was available for its subjects. A longitudinal study should also be conducted in the future. In addition, the similarities and differences between AD-associated and healthy aging patterns merit further study. Finally, although we chose the entire cerebellum as the reference region for calculating the SUVR values, the selection of the reference region has long been a methodologically challenging issue within studies entailing PET imaging analysis. Future comparative studies of different reference regions are urgently required to develop a comprehensive understanding of this compound.

## Conclusion

In summary, we performed a voxel-wise correlation analysis to identify a pattern associated with changes in β-amyloid deposition in cognitively normal adults during healthy aging. An assessment of the pattern advances understanding of processual changes entailed in brain aging. The changes in Aβ deposition associated with healthy aging that are reflected in age-associated longitudinal changes of Aβ deposition on specific brain regions are indicative of opportunities for diagnosis and strategies for decelerating aging. More generally, this study may reveal a pattern of changes in Aβ deposition that can be used to distinguish physiological changes from pathophysiological ones.

## Data Availability Statement

The datasets presented in this study can be found in online repositories. The names of the repository/repositories and accession number(s) can be found in the article/supplementary material.

## Ethics Statement

The studies involving human participants were reviewed and approved by the institutional review boards of ADNI and the Research Ethics Committee of Xuanwu Hospital, Beijing, China. The patients/participants provided their written informed consent to participate in this study.

## Author Contributions

YX and QY are responsible for collecting data, designing experiment and writing article. CL and QZ are responsible for the experimental coding. JJ and YH are responsible for the guidance of the experiment and paper. All authors contributed to the article and approved the submitted version.

## Conflict of Interest

The authors declare that the research was conducted in the absence of any commercial or financial relationships that could be construed as a potential conflict of interest.
